# Unveiling Place-Based Effects at Scale: A Multiscale Geographically Weighted Regression of Food Deserts and Cardiovascular Risk in Chile

**DOI:** 10.3390/epidemiologia7020042

**Published:** 2026-03-10

**Authors:** Francisco Vergara-Perucich, Leslie Landaeta-Díaz, Carlos Aguirre-Nuñez

**Affiliations:** 1 Núcleo de Investigación Centro Producción del Espacio, Universidad de Las Américas, Manuel Montt 948, Santiago 7500975, Chile; 2 Núcleo de Investigación en Nutrición y Ciencias Alimentarias, Universidad de Las Américas, Manuel Montt 948, Santiago 7500975, Chile; llandaeta@udla.cl; 3 Escuela de Arquitectura, Universidad San Sebastián, Lota 2465, Santiago 8420524, Chile; carlos.aguirre@uss.cl

**Keywords:** food environment, food deserts, environmental epidemiology, spatial epidemiology, Chile

## Abstract

Background/Objectives: Cardiovascular diseases (CVD) in Chile are profoundly shaped by place-based determinants of diet. This study examines the association between food deserts—areas with structurally limited access to nutritious, affordable food—and population-level cardiovascular risk across Chile’s three largest metropolitan areas (Santiago, Valparaíso, Concepción). Methods: We constructed a geospatial food desert index combining OpenStreetMap-derived retail accessibility with census information, and linked it to georeferenced cardiovascular health records. To overcome the limitations of global models that assume spatial stationarity, we applied Multiscale Geographically Weighted Regression (MGWR) to allow coefficients to vary across space and to recover variable-specific process scales. Results: The MGWR results indicate pronounced spatial non-stationarity in the food desert–CVD association. The relationship is predominantly positive across Gran Valparaíso, predominantly negative in Gran Concepción, and highly mixed within Gran Santiago, evidencing divergent local mechanisms rather than a single national pattern. Conclusions: The observed heterogeneity undermines “one-size-fits-all” national interventions and supports place-sensitive, equity-oriented strategies. Policy implications include territorially tailored food-retail regulation and primary-care outreach, co-designed with local actors, with MGWR providing a critical analytic basis for actionable, context-specific public health planning.

## 1. Introduction

Cardiovascular diseases (CVD) persist as the leading cause of mortality globally, a burden increasingly understood not merely as a product of individual genetics or behaviour, but as a manifestation of complex, place-based determinants. Environmental epidemiology, while traditionally focused on discrete physicochemical pollutants, is undergoing a critical paradigm shift [1,2,3]. This shift recognizes the built environment—the very fabric of our cities—as a pervasive and chronic exposure, one that systematically shapes behaviours, resource access, and long-term health trajectories. Within this framework, the urban food environment emerges as a critical domain, structurally mediating nutritional access and directly influencing the global epidemic of non-communicable diseases (NCDs). This is particularly salient in low- and middle-income countries (LMICs) like Chile, where rapid and often inequitable urbanization compounds these risks, creating stark new geographies of health inequality [4].

This study explicitly positions the urban food environment as a consequential environmental exposure. We conceptualize access to nutrition not primarily as a matter of individual choice, but as a condition structurally determined by urban form, market dynamics, and public policy. The concept of the “food desert”—an area with structurally limited access to affordable, nutritious food—serves as an operational definition for a harmful environmental exposure, one that disproportionately affects socioeconomically vulnerable populations [5,6]. The persistent scarcity of healthy food retailers, often coupled with a high density of outlets promoting energy-dense, nutrient-poor products, constitutes a potent obesogenic exposure that fuels cardiometabolic risk. By framing food access in this way, we can apply the rigorous toolkit of environmental epidemiology to a key driver of health disparities, shifting the discourse from individual responsibility toward the principles of environmental justice.

However, quantifying the health impact of this complex exposure presents a formidable methodological challenge. The prevailing research paradigm has heavily relied on “global” statistical models, such as standard linear regression, which operate under the assumption of spatial stationarity—the notion that the relationship between an exposure and an outcome is constant across space. This assumption represents a profound over-simplification in the context of a heterogeneous, segregated metropolis. The effect of a food desert on cardiovascular health in a dense, low-income urban core is unlikely to be identical to its effect in a sprawling, car-dependent suburb. Ignoring this spatial non-stationarity risks producing misleading, averaged-out conclusions that, when translated into policy, yield ineffective “one-size-fits-all” interventions incapable of addressing the specific needs of diverse communities.

Soto et al. documented an overall decline in CVD mortality from 159.5 to 94.6 per 100,000 population between 2000 and 2020 [7]. Lavados et al. found a twofold increase in stroke mortality rates across different regions, with 62% of regional variability explained by factors like poverty (34%), diabetes (17%), sedentary lifestyle (8%), and overweight (3%) [8]. Flores et al. identified key structural determinants, including race, sex, socioeconomic level, and educational level, with low socioeconomic and educational levels being most strongly associated with CVD incidence [9]. Sánchez and Albala further confirmed a negative correlation between income and mortality, particularly among men [10,11].

To overcome this limitation, our study employs an advanced spatial econometric technique: Multiscale Geographically Weighted Regression (MGWR) [12]. As a significant evolution of Geographically Weighted Regression (GWR) [13], MGWR moves beyond the restrictive assumption of a single, uniform spatial scale for all processes. Its key innovation is the calibration of a unique, optimal bandwidth for each predictor variable, acknowledging that different social and environmental processes operate at different spatial scales [12,14]. This allows us to model, for instance, whether the influence of the local food retail environment is a hyper-local, neighbourhood-level phenomenon, while the impact of broader regional socioeconomic factors operates at a larger scale. This methodological refinement is indispensable for constructing a more realistic and interpretable model of the complex exposure mixtures that constitute the urban environment, offering a superior approach for diagnosing spatial heterogeneity [15,16].

This paper applies the MGWR framework to a critical case study: Chile’s three largest metropolitan areas (Greater Santiago, Valparaíso, and Concepción). By linking a novel geospatial indicator of food deserts—constructed from OpenStreetMap and census data—to georeferenced cardiovascular risk records from a national health programme, we directly quantify the spatially varying association between food desert exposure and population-level CVD risk [5]. Our objectives are twofold and reflect the dual substantive and methodological contribution of our work. First, we provide a robust empirical demonstration of the marked spatial non-stationarity in this relationship, testing the core hypothesis that the food desert–CVD link is not uniform but is instead contingent on local context [5,12,16]. Second, we showcase the analytical power of MGWR as a next-generation tool for spatial epidemiology, capable of yielding policy-relevant, territorialised insights into the multiscale nature of place-based health determinants [12,14,15].

The findings, which reveal a spectrum of associations from strongly positive in Valparaíso to predominantly negative in Concepción and highly mixed in Santiago, fundamentally challenge the logic of uniform policy approaches. By rigorously operationalizing retail access as a quantifiable exposure, this study contributes to the advancing frontiers of environmental epidemiology. It underscores the urgent need for place-sensitive, equity-oriented public health strategies that are co-designed with municipal and community actors. Ultimately, our work challenges policymakers and researchers to move beyond simplistic, national-level targets and to embrace the intricate geographical complexity of health, fostering a more nuanced dialogue on preventive strategies that can mitigate the health risks embedded in our built environments.

### Literature Review

Cardiovascular diseases (CVD) are the leading cause of premature mortality globally [17,18]. Suboptimal diet and nutrition are primary modifiable risk factors contributing to global morbidity [17]. This crisis is situated within the Global Syndemic, defined by the concurrent existence of undernutrition, obesity, and nutrition-related chronic diseases (NRCDs) [16,19].

The academic field dedicated to studying these links, nutritional epidemiology, has faced severe criticism for its historical reliance on relatively weak observational study methods and substandard measurements [3]. These methodological deficiencies frequently lead to unwarranted claims of causality [3], fostering scepticism amongst both the public and experts [20]. Some critics have gone as far as suggesting the field should be abandoned, arguing that its observational nature remains fundamentally limited [3,12,21]. However, a more productive consensus advocates for reform, urging scholars to reject the notion that good enough is no longer good enough [3]. This requires greater scientific rigour, the adoption of stronger designs (including quasi-experimental methods), enhanced objective measurement, and greater transparency through preregistration and core outcome sets [3]. Even highly complex dietary studies, such as those examining vegetarianism and cancer, often yield contradictory results due to the intricacy of the topic, emphasising the need for methodological strength [4]. Contemporary tools, such as those used in Big Epidemiology [2], offer new avenues for analysing large datasets, yet the fundamental requirement remains high-quality evidence linking the environmental exposome to health outcomes [3].

The geographical environment is not a passive backdrop for health outcomes; it is an active agent which shapes health and opportunity [4]. Urban planning and public health, historically linked disciplines, must be reunited to understand the ecology of food environments as a key social determinant of health [4,10]. The built environment, including the neighbourhood context, is a critical domain for chronic disease development, comparable to other factors such as economic stability [13].

The core manifestation of nutritional inequity is the unequal distribution of retail, creating food deserts (areas with poor access to healthy options) and food swamps (areas dominated by ultra-processed food outlets) [17,22,23].

Multiple pieces of empirical evidence confirm that spatial exposure to unhealthy environments significantly mediates and modifies chronic disease risk:

Ultra-Processed Food Risk: In Brazilian municipalities, a greater availability (density) of ultra-processed foods generally increased the risk of premature mortality from CVDs, strokes, and Ischaemic Heart Disease (IHD) [17]. Conversely, a lower availability of unprocessed foods increased the risk of heart attack mortality in women [17].

Socioeconomic Concentration: The adverse effect of living in a food desert on cardiovascular health is concentrated amongst individuals of low socioeconomic status (SES) [22]. This is described as the amplification of the deprivation, where limited economic resources heighten susceptibility to environmental hazards [22].

Geographical Inequality in LMICs: Systematic reviews confirm that in Low- and Middle-Income Countries (LMICs), particularly in urban settings, there is consistent evidence linking availability characteristics in the neighbourhood food environment to dietary behaviour [24]. However, high-quality analytical studies are scarce, and most evidence stems from Upper-Middle-Income Countries (UMICs), leaving a substantial evidence gap for Low-Income Countries (LICs) [19,24]. Differences in findings between LMICs and High-Income Countries (HICs) may stem from socioeconomic factors and differences in individual mobility [24].

Complex Urban Patterns: Analysis of Santiago, Chile, demonstrated that food deserts were spatially concentrated in low SES peripheral areas, yet paradoxically, they were also found in certain high SES central areas. This complexity suggests that food inequity is driven by multifaceted issues, including car dependence and urban design, which are profound structural inequalities inscribed upon the territory [6,14].

Interventions focused on changing the built environment, such as introducing new supermarkets in underserved areas, have often yielded null effects on outcomes like BMI or fruit/vegetable consumption [6,23,25]. While residents often perceive a significant improvement in the quality of their neighbourhood food environment following such interventions, this does not always translate into improved dietary behaviour [6]. This outcome highlights that merely addressing availability (a domain of the food environment) is insufficient; additional strategies, such as subsidised prices or in-store marketing, may be needed [23]. Policy efforts must move beyond simplistic approaches [17] towards holistic strategies that support sustained behavioural changes, such as integrating nutrition into home visiting programmes [26]. The overarching goal is to achieve the affordability of healthy diets and design environments that do not impose disproportionate costs of time or mobility [6].

To translate social injustices into legible geographical patterns and inform policy [4], a rigorous methodological framework is essential. Spatial epidemiology, using tools like Geographical Information Systems (GIS) [1,9], allows for the modelling of environmental exposures and the analysis of spatial distribution patterns in disease [5]. The discipline must move towards robust, replicable, and scalable evidence [27].

A fundamental challenge in spatial modelling is spatial nonstationarity, where the relationship between predictors and outcomes varies geographically [8]. While Geographically Weighted Regression (GWR) addresses nonstationarity by calibrating local parameters, it imposes the restriction that all processes operate at a single spatial scale, defined by a single optimal bandwidth [7,8]. This can lead to model misspecification when dealing with multi-factorial processes, such as those contributing to obesity, which operate across multiple scales [16].

Multiscale Geographically Weighted Regression (MGWR) overcomes this constraint. MGWR calibrates the model using a back-fitting algorithm to derive an optimal bandwidth vector, where each element identifies the specific spatial scale at which a particular predictor variable operates [8,15]. This provides valuable and intuitive information regarding process scale, and MGWR has been demonstrated to be superior to GWR in replicating parameter surfaces with varied spatial heterogeneity [8]. This methodological advance is indispensable for identifying precisely whether a food desert’s effect is hyper-local (a small bandwidth) or more regional (a large bandwidth) [16].

The implementation of MGWR necessitates strict adherence to contemporary best practices to ensure robustness [16]. This includes investigating local multicollinearity, and, crucially, accounting for parameter uncertainty. The estimation of a bandwidth, even in MGWR, is typically treated as deterministic [16]. However, the uncertainty inherent in bandwidth selection must be measured, for instance, using Akaike Weights, as ignoring this uncertainty can lead to an underestimate of the variance for local parameter estimates, making results appear spuriously significant [15]. The corrected Akaike Information Criterion (AICc) is preferred for bandwidth selection, particularly where the ratio of data points to parameters is low [15].

By applying MGWR, researchers can move beyond descriptive diagnosis towards robust policy evaluation, providing territorialisable evidence that permits decision-makers to implement finely tuned policies, such as economic instruments or tax measures against unhealthy foods [17], thereby achieving fairness in urban design [27]. The rigorous application of these advanced methods is particularly vital in the context of LMICs, where comprehensive systematic review evidence on the food environment remains sparse, primarily due to heterogeneity in exposure definition and measurement [18].

## 2. Materials and Methods

### 2.1. Study Design and Scope

**Figure 1:**
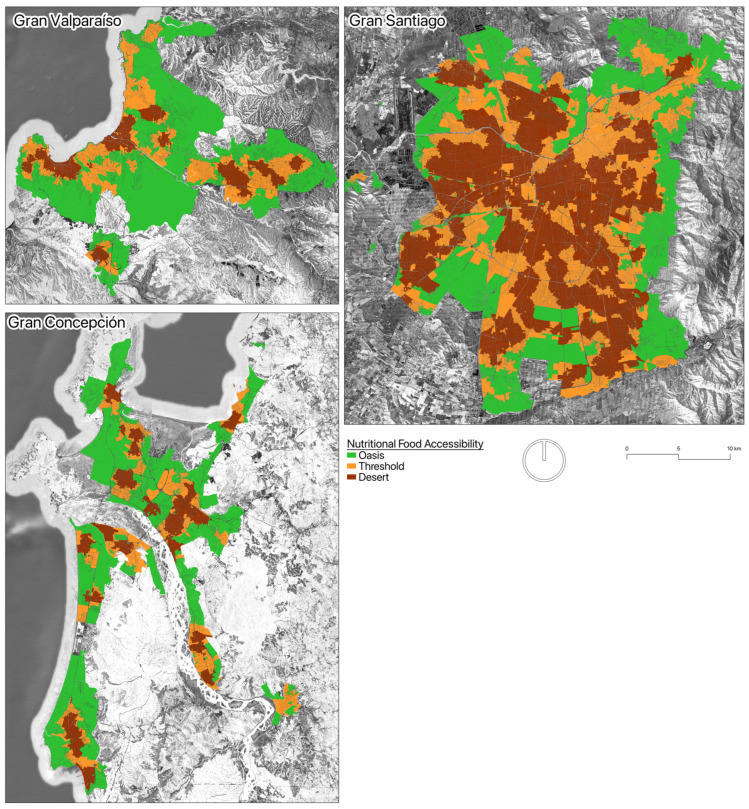
Food deserts in Chilean metropolitan areas.

This study employs a cross-sectional, ecological design to investigate the spatially varying relationship between the urban food environment and cardiovascular risk. The analysis is focused on Chile’s three largest metropolitan areas: Gran Santiago (the national capital), Gran Valparaíso (a coastal port conurbation), and Gran Concepción (an industrial, river-based city) (Figure 1). These conurbations were selected due to their demographic significance, their diverse urban morphologies, and the availability of sufficiently dense data to support robust local statistical modelling. By examining these distinct urban systems within a single middle-income country context, we aim to provide a nuanced, comparative analysis of how place-based environmental exposures shape health outcomes. The unit of analysis is the census zone, allowing for a high-resolution, sub-municipal examination of spatial patterns.

### 2.2. Data Sources and Variable Definitions

The analysis relies on two primary georeferenced datasets compiled for the FONIS-ANID SA23I0032 project.

- Food Desert Index (Independent Variable): This variable operationalizes the concept of a food desert as an environmental exposure. It is a continuous index ranging from 0 (indicating good access to healthy food, or a food oasis) to 1 (indicating poor access, or a food desert). The index was constructed through a spatial analysis of food retail locations derived from OpenStreetMap and population data from the 2017 Chilean Census, reflecting the structural availability of healthy food options relative to population density.
- Cardiovascular Risk Rate (Dependent Variable): The health outcome is represented by the rate of patients enrolled in the national Cardiovascular Health Programme (Programa de Salud Cardiovascular, PSCV) per census zone. This variable serves as a proxy for diagnosed cardiovascular morbidity within the population. The data was obtained from official Ministry of Health records and georeferenced to the corresponding census zones, providing a spatially explicit measure of cardiovascular risk.

### 2.3. Statistical Analysis

The central objective of this paper is to move beyond the flawed assumption of spatial stationarity inherent in global regression models. To achieve this, our analytical workflow is structured in sequential stages.

Stage 1: Global Diagnostics. As a preliminary step, we conducted a global analysis to establish a baseline and justify the need for local models. A standard multiple linear regression was performed at the national level to model the food desert index as a function of the PSCV rate, a 15-min city score, and the type of health provider. Subsequently, we calculated the Global Moran’s I statistic for both the food desert index and the PSCV rate to formally test for spatial autocorrelation. A k-nearest neighbours (k = 8) spatial weights matrix was constructed to define the neighbourhood structure for this test. The finding of significant spatial clustering in the food desert index (I = 0.4563, *p* < 2.2 × 10^−16^) invalidated the independence assumption of non-spatial models and confirmed the necessity of spatially explicit techniques.

Stage 2: Multiscale Geographically Weighted Regression (MGWR). The core of our analysis is the application of Multiscale Geographically Weighted Regression (MGWR) to the three metropolitan areas. We selected this advanced method over standard Geographically Weighted Regression (GWR) because of its unique ability to model processes that operate at different spatial scales. While GWR assumes a single bandwidth for all relationships, MGWR estimates an optimal, variable-specific bandwidth for each predictor, which is crucial for obtaining a more realistic and interpretable model of the urban environment.

For this study, we specified a bivariate MGWR model: Rate of CVD~ Food Desert. The model was calibrated using an adaptive bisquare kernel, which allows the neighborhood size to vary based on data density, and bandwidths were optimized using the corrected Akaike Information Criterion (AICc) to balance model fit and complexity. The output is a continuous surface of local regression coefficients for each census zone, which is then mapped to visualize how the strength and direction of the association between food desert exposure and cardiovascular risk changes across the urban landscape. All analyses were conducted using the R statistical programming language, primarily with the spdep, sf, and GWmodel packages.

### 2.4. Limitations

This study has several important limitations. First, its cross-sectional, ecological design identifies statistical associations and cannot establish causality; the findings are subject to the ecological fallacy. The observed relationships could be influenced by numerous unmeasured confounding variables at the individual level, such as lifestyle factors, income, or residential self-selection. Second, the analysis is subject to the Modifiable Areal Unit Problem (MAUP), meaning the results could be sensitive to the specific boundaries of the census zones used as the unit of analysis. Third, data availability restricted the robust application of MGWR to only three metropolitan areas. Finally, the PSCV data serves as a proxy for diagnosed morbidity and may be influenced by differential access to healthcare and reporting practices, which could introduce measurement error and partially explain some of the counterintuitive findings, as suggested by the global regression results. Despite these limitations, the study’s primary strength lies in its explicit focus on modeling spatial heterogeneity, providing a powerful demonstration of why place-sensitive, context-specific approaches are essential for effective public health policy.

## 3. Results

This section details the statistical findings of the study, progressing from a national-level global analysis to a spatially explicit local analysis of the relationship between food deserts and cardiovascular risk in Chile’s three main metropolitan areas.

### 3.1. Global-Scale Associations: National Regression Model

To establish a national baseline, a multiple linear regression model was fitted to identify factors associated with the presence of food deserts across Chile. The model, which was statistically significant (F-statistic = 9.586, *p* < 0.001), explained approximately 16% of the variance (Adjusted R^2^ ≤ = 0.1602) in the food desert index2. The dependent variable was the Food Desert Index (0 = no desert, 1 = desert), and the predictors included the rate of patients in cardiovascular health programs (PSCV), the 15-min city score, and the type of local health provider. The estimated coefficients are presented in Table 1.

Two factors emerged as statistically significant predictors at the national level:

15-Minute City Score: This was the strongest predictor. The model indicates a robust negative association (E ≤ −0.0174), suggesting that for every one-point increase in the urban accessibility score, the probability of an area being a food desert decrease by 1.74 percentage points. This aligns with the expectation that better-equipped neighbourhoods are less prone to food insecurity.

PSCV Rate: A significant but counterintuitive negative relationship was found (E ≤ −0.0036). The model suggests that higher rates of cardiovascular patients are associated with a slightly lower probability of an area being a food desert8. This unexpected finding may reflect complex confounding factors, such as the possibility that areas with better health infrastructure (and thus fewer food deserts) are also more effective at diagnosing and reporting cardiovascular cases. The type of health provider (public vs. private) showed no significant association.

### 3.2. Spatial Distribution Patterns: Moran’s I Analysis

To formally test for spatial patterns, a global spatial autocorrelation analysis using Moran’s I statistic was performed on the PSCV rates and the food desert index. This step is crucial to determine whether the phenomena are geographically clustered, which would justify the use of local spatial models. The results are summarized in Table 2.

The analysis revealed two distinct spatial realities:

PSCV Rates: The Moran’s I statistic was very close to zero (0.0221) and was not statistically significant (*p* = 0.190). This indicates that, at a national scale, the geographic distribution of cardiovascular patient rates is consistent with a random pattern.

Food Deserts: In stark contrast, the food desert index showed a strong and highly significant positive spatial autocorrelation (I = 0.4563, *p* < 2.2 × 10^−16^). This result provides compelling evidence that food deserts are not randomly scattered; rather, they are systematically grouped together, forming distinct geographical clusters of nutritional disadvantage. This finding validates the need for spatially explicit local analyses.

### 3.3. Local Heterogeneity: Multiscale Geographically Weighted Regression (MGWR)

Given the evidence of significant spatial clustering, an MGWR analysis was conducted to explore how the relationship between the food desert index and the PSCV rate varies locally. This analysis was robustly performed in the three metropolitan areas with sufficient data density: Gran Valparaíso, Gran Concepción, and Gran Santiago. The model Rate of CVD~ Food Desert was fitted to assess whether living in a food desert is associated with higher PSCV rates at the local level.

#### 3.3.1. Gran Valparaíso

In Gran Valparaíso, the MGWR analysis (Table 3) revealed a clear and consistent positive association between food deserts and cardiovascular risk across the entire metropolitan area.

The local coefficients were positive everywhere, ranging from 5.007 to 21.279, with a median of 19.65. This indicates that in Valparaíso, areas with a higher food desert index are consistently and strongly associated with higher rates of patients in cardiovascular programs, supporting the hypothesis of a direct negative link between poor food access and cardiovascular health in this region.

#### 3.3.2. Gran Concepción

The results for Gran Concepción (Table 4) presented a strikingly different and counterintuitive picture. The relationship was found to be predominantly negative.

The median local coefficient was −13.51, and the range of coefficients was largely negative (−54.045 to 4.233). This suggests that in most parts of Concepción, a higher food desert index is unexpectedly associated with lower PSCV rates. While the overall trend is negative, the fact that the range crosses zero indicates that there are small pockets where the relationship could be null or even positive.

#### 3.3.3. Gran Santiago

As the largest and most complex metropolis, Santiago exhibited the highest degree of spatial heterogeneity (Table 5).

The analysis revealed a mixed and highly variable relationship that does not follow a single pattern. The local coefficients ranged from negative to positive (−5.627 to 1.067), confirming that the association between food deserts and cardiovascular health changes significantly from one sector of the city to another. In some municipalities, the expected positive association may hold, while in others, the relationship is null or even inverted, likely due to the complex interplay of socioeconomic status, mobility infrastructure, and local health programs. This heterogeneity itself is a key finding, demonstrating that a uniform public policy for a city as diverse as Santiago would likely be ineffective.

## 4. Discussion

This study set out to investigate the relationship between food deserts, as a place-based environmental exposure, and cardiovascular risk across three distinct Chilean metropolises. The findings reveal spatial heterogeneity, fundamentally challenging the notion of a uniform, one-size-fits-all public policy approach to nutritional health. By moving beyond the assumption of spatial stationarity, our results not only confirm the importance of the urban food environment but also illuminate the complex, context-dependent nature of its impact, providing a spatially explicit explanation for why simple interventions may fail and offering a path toward more precise, equitable public health strategies.

The analysis of Gran Valparaíso yielded a clear and consistently positive association, aligning with the food desert hypothesis. In this region, areas with poorer access to healthy food are directly and strongly associated with higher rates of cardiovascular morbidity. This finding empirically supports literature linking exposure to unhealthy food environments with premature CVD mortality [17] and underlines the concept of deprivation amplification, where the adverse health effects of food deserts are most concentrated among vulnerable populations [22]. For urban contexts that mirror Valparaíso’s direct positive correlation, policies aimed at improving the physical availability of nutritious food appear to be justified and high-priority interventions.

In stark contrast, the predominantly negative and mixed associations found in Gran Concepción and Gran Santiago, respectively, present a more complex narrative. These counterintuitive results do not necessarily refute the detrimental effect of food deserts. Instead, they provide spatial evidence for the multifaceted mechanisms suggested in the literature. One plausible explanation, hinted at by our global regression model, is confounding by healthcare access and data capture. Areas with better food access may also possess more robust health systems, leading to higher rates of diagnosed and reported cardiovascular disease. This would create a spurious negative correlation where better environments appear to have worse health outcomes.

Furthermore, these findings resonate with literature highlighting the limitations of simplistic interventions. Studies showing that the introduction of a new supermarket often has null effects on dietary behaviour and BMI underscore that availability is only one piece of a complex puzzle [23,25]. Our results provide a geographical basis for these null effects: if the relationship between local food access and health is negative or non-existent in large parts of a city, then simply adding a new food source is unlikely to succeed without addressing other, more powerful determinants. These may include individual mobility patterns, which are known to differ between high-income and low- and middle-income country contexts [24], or complex urban patterns—such as car dependency—that can drive food inequity even in high-income areas [5].

From a methodological standpoint, this study serves as a proof of the need for advanced spatial techniques in nutritional epidemiology, responding directly to calls for greater scientific rigour in the field [3]. By employing Multiscale Geographically Weighted Regression (MGWR), we move beyond the restrictive single-bandwidth assumption of traditional GWR, which can misspecify models when dealing with multifactorial processes operating at different spatial scales [12,16]. MGWR functions here as a diagnostic device, revealing where a particular relationship holds and where it breaks down. This capacity to produce territorialisable evidence is precisely what is needed to translate social injustices into legible geographical patterns that can inform policy [27].

The implications for public health policy are clear. The era of uniform, national-level strategies for tackling food environments must give way to a place-sensitive, data-driven approach. In a city like Valparaíso, direct interventions on food supply are warranted. In Concepción, the immediate priority should be to investigate potential confounding by healthcare data systems. In Santiago, interventions must be hyper-local—designed at the municipal or even neighbourhood scale—and tailored to the specific drivers of inequity in each context. Ultimately, our findings advocate a holistic strategy that moves beyond merely addressing availability toward policies that ensure the affordability of healthy diets in environments that do not impose disproportionate costs of time or mobility [17,27]. Future research should build on this work with longitudinal designs to better probe causality and integrate individual-level mobility data to refine our understanding of environmental exposure.

## 5. Conclusions

This study has provided a high-resolution, spatially explicit analysis of the relationship between food deserts and cardiovascular risk in urban Chile, revealing a landscape of profound geographical complexity. By framing the food environment as a modifiable environmental exposure and applying advanced spatial methods, our findings move beyond simplistic, universal assumptions to offer a more nuanced understanding of how place shapes health. The core contribution of this work lies not in identifying a single, uniform effect, but in demonstrating that the mechanisms linking food accessibility to cardiovascular health are intrinsically context-dependent, demanding a fundamental shift in how we design and implement public health policy.

The divergent results from Chile’s three largest metropolises tell a tale of three cities. In Gran Valparaíso, the consistent positive association between food deserts and cardiovascular risk aligns with the classic environmental health hypothesis, providing strong justification for interventions that directly improve the availability of healthy food. However, the predominantly negative and mixed results in Gran Concepción and Gran Santiago, respectively, challenge this straightforward narrative. These counterintuitive findings do not necessarily negate the harmful effects of poor food access; rather, they illuminate the critical role of confounding factors and the limitations of ecological study designs. They suggest that in some urban contexts, variables such as differential access to healthcare, the quality of health data reporting, and complex mobility patterns may obscure or even invert the expected relationship at the aggregate level. This underscores a key lesson for environmental epidemiology: the absence of a simple, direct association does not mean the absence of a problem but rather points to a more complex causal web that requires deeper investigation.

Methodologically, this study serves as a robust proof of concept for the application of Multiscale Geographically Weighted Regression (MGWR) in nutritional and environmental epidemiology. The technique proved indispensable for moving beyond the flawed assumption of spatial stationarity, providing a diagnostic stethoscope to listen to the unique health dynamics of different urban territories. The ability of MGWR to reveal where a statistical relationship holds, where it weakens, and where it inverts transforms it from a mere analytical tool into a powerful instrument for policy design. It provides the territorialisable evidence necessary to avoid the pitfalls of “one-size-fits-all” solutions, which are likely to fail in the face of such marked local heterogeneity.

The policy implications stemming from these findings are clear and urgent. The evidence strongly refutes the efficacy of monolithic, top-down national strategies and calls for a paradigm shift toward place-sensitive and adaptive public health governance. Interventions must be tailored to the specific spatial signature of each urban area: direct retail and access-based policies may be effective in cities like Valparaíso, whereas in places like Concepción, the priority might first be to audit and improve health data systems. For a complex mosaic like Santiago, a hyper-local approach, co-designed with municipal and community actors who understand the unique barriers and assets of their neighborhoods, is essential.

Ultimately, this research makes a case for a more geographically intelligent and ethically conscious form of public health. The maps of nutritional inequality are, in their essence, maps of environmental injustice. By making these patterns legible, spatial epidemiology provides the evidence needed to hold systems accountable and to design interventions that are not only effective but also equitable. The future of the field lies in building upon this work through longitudinal studies, integrating individual-level mobility data to better measure exposure, and applying these advanced methods in other LMIC contexts. This shift is not merely methodological but normative; it demands that we recognize the city as an active determinant of health and use the tools of spatial science to begin redesigning it for justice.

## Figures and Tables

**Table 1:** Coefficients of the Global Linear Regression Model.

Variable	Estimate	Std. Error	t-Value	*p*-Value
(Intercept)	0.837	0.10862	7.706	<0.001
PSCV Rate	−0.00364	0.00161	−2.267	0.0243
15-Min City Score	−0.01743	0.00275	−6.331	<0.001
Provider: PRIVATE	−0.15794	0.11235	−1.406	0.1612
Provider: PUBLIC	−0.10311	0.10867	−0.949	0.3437

**Table 2:** Global Moran’s I Test for Spatial Autocorrelation.

Variable	Moran’s I Statistic	Expected Value	*p*-Value	Interpretation
PSCV Rate	0.0221	−0.0044	0.19	Random
Food Desert	0.4563	−0.0044	<2.2 × 10^−16^	Clustered

**Table 3:** MGWR Results for Gran Valparaíso.

Model	Independent Variable	Global Coefficient	Median Local Coefficient	Range of Local Coefficients	Relationship
MGWR 2	Food Desert	16.37	19.65	5.007 to 21.279	Positive

**Table 4:** MGWR Results for Gran Concepción.

Model	Independent Variable	Global Coefficient	Median Local Coefficient	Range of Local Coefficients	Relationship
MGWR 2	Food Desert	−35.56	−13.51	−54.045 to 4.233	Predominantly Negative

**Table 5:** MGWR Results for Gran Santiago.

Model	Independent Variable	Global Coefficient	Median Local Coefficient	Range of Local Coefficients	Relationship
MGWR 2	Food Desert	−1.03	−3.34	−5.627 to 1.067	Mixed/Variable

## Data Availability

Data supporting the findings of this study are available upon reasonable request to the corresponding author. The study relied on secondary data sources, ensuring no ethical issues in data collection.
